# Association between macrophage migration inhibitory factor promoter region polymorphism (-173 G/C) and cancer: a meta-analysis

**DOI:** 10.1186/1756-0500-4-395

**Published:** 2011-10-11

**Authors:** Pedro L Vera, Katherine L Meyer-Siegler

**Affiliations:** 1 The Bay Pines VA Healthcare System, Research & Development, Bay Pines, Florida, USA; 2 University of South Florida, Department of Surgery, Division of Urology, Tampa, Florida, USA; 3 University of South Florida, Department of Molecular Medicine, Tampa, Florida, USA

## Abstract

**Background:**

Macrophage migration inhibitory factor (MIF) is a pro-inflammatory cytokine upstream of many inflammatory cytokines. MIF is implicated in several acute and chronic inflammatory conditions. MIF's promoter region has functional single nucleotide polymorphisms that controls MIF expression and protein levels. Since increased plasma MIF levels are associated with cancer, studies have examined the association between *Mif *promoter polymorphisms and cancer. This study is a meta-analysis of the available studies on such an association.

**Results:**

A total of 5 studies were included in this meta-analysis to include 1116 cases (cancer patients) and 1728 controls (no cancer). Carrying any C allele in the *Mif *-173 G/C promoter polymorphism resulted in a significantly greater risk for developing cancer [OR = 1.89 (1.15-3.11), p = 0.012)] when compared to the (G/G) genotype. Subgroup analysis revealed that this association was significant only for "solid" tumors (including gastric and prostate cancers) [OR = 2.67 (1.26-5.65), p = 0.010] but not for "non-solid" tumors (leukemia) [OR = 1.21 (0.95-1.55), p = 0.122]. Furthermore, when only prostate tumor studies were included in the analysis, the association became even stronger [OR = 3.72 (2.55-5.41), p < 0.0001].

**Conclusions:**

Meta-analysis suggests there is an association between any C allele in the *Mif *-173 G/C promoter polymorphism and an increased risk of cancer, particularly for solid tumors. The association appeared stronger for prostate cancer, specifically. Future studies that include different types of cancers are needed to support and extend these observations.

## Background

Macrophage migration inhibitory factor (MIF) was the first cytokine identified nearly 50 years ago. At that time its described activity was as a soluble factor produced by T-lymphocytes that inhibited the directed migration of macrophages [1]. Since then, MIF is documented to be expressed by a broad variety of cells and tissues, including immune and non-immune cells [2,3]. Presently, MIF is considered a pleiotropic cytokine that is a central regulator of innate immunity and a regulator of inflammation since it is constitutively expressed and acts as an upstream regulator of many other inflammatory cytokines [4]. Experimental evidence has shown that MIF plays a critical role in immune and inflammatory diseases including sepsis [5], rheumatoid arthritis [6], delayed-type hypersensitivity [7], Crohn's disease [8] and gastric ulcer formation [9].

Because MIF is an important regulator of inflammation and given the established link between chronic inflammation and cancer [10], recent studies have examined the link between *Mif *mRNA expression and/or MIF protein levels and cancer [11]. Increased *Mif *mRNA expression in prostate tissue [12] and increased serum MIF protein levels [13] were first documented for prostate cancer patients. Other investigations corroborated these findings for prostate cancer [14,15] and extended the association between increased *Mif *mRNA and/or protein expression to other types of cancer [16-19]. Therefore, strong evidence has accumulated suggesting that MIF is an important link between inflammation and cancer [11]. Two regions within the *Mif *promoter contain functional polymorphisms that result in variable *Mif *mRNA amounts and subsequent protein production and high expressing promoter polymorphism genotypes are associated with increased risk of inflammatory disease [20,21]. A -173 G/C transversion results in a single nucleotide polymorphism (SNP) that was first described with higher frequency in arthritis patients [22,23]. Another functional promoter polymorphism was identified at position -794, where increasing copies of a CATT tetranucleotide repeat (5 to 8 copies, -794 CATT(5-8)) were also associated with increased disease severity in arthritis [20]. The association between these polymorphisms and disease has been extended to other inflammatory conditions including ulcerative colitis [24], peptic ulcer disease [25] and increased morbidity due to sepsis [26]. These studies thus suggest that an association exists between *Mif *promoter genotypes that result in increased MIF protein production and an increased risk of inflammatory disease. Similarly, studies examining the association between *Mif *promoter polymorphisms and cancer have reported an increased risk of cancer for patients with higher MIF protein producing promoter genotypes [3,27-30] while others have reported a lack of association [31]. The aim of this study is to conduct a meta-analysis of all available studies that examine the association between *Mif *promoter polymorphism and the incidence of cancer.

## Results

### Studies Eligibility

After a literature search and selection based on inclusion criteria as described in the methods, five studies were identified for meta-analysis [27-31]. Table 1 lists these studies and their characteristics. The data for the meta-analysis included 1,116 cancer cases and 1,728 controls (no cancer). *Mif *-173 G/C polymorphism groups were divided as G/G (control comparison) and any C/X (i.e. G/C or C/C). All studies were published between 2005 and June 2011. Two studies were conducted on leukemia patients and were thus classified as "non-solid tumors" whereas the remaining three studies examined either gastrointestinal or prostate tumors and thus were classified as "solid tumors".

**Table 1:** Studies included in meta-analysis

Studies	Year	Cases (Cancer)		Controls (No cancer)		Tumor Type
		***Any C***	***G/G***	***Any C***	***G/G***	

Ziino	2005	34	117	78	277	Leukemia

Meyer-Siegler	2007	76	55	29	99	Prostate

Arisawa	2008	106	123	167	261	Gastric

Ding	2008	93	166	45	256	Prostate

Xue	2010	110	228	147	369	Leukemia

Studies were selected from published articles gathered from a search of the literature using PubMed and meeting the selection criteria (as listed in Methods).

### Meta-analysis results

**Figure 1:**
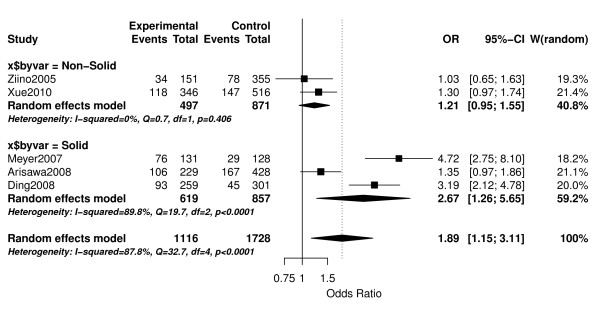
**Meta-analysis of *Mif *promoter region polymorphism (-173 G/C) and cancer**. Point estimates of the Odds Ratio (OR) and accompanying 95% confidence intervals are shown for each study. Overall analysis using Random effects model and subgroup analyses for patients with "Non-Solid" vs. "Solid" tumors are shown.

Meta-analysis of the 5 eligible studies showed that individuals that carry any C allele in the *Mif *promoter region at position -173 had a significantly higher risk for developing cancer when compared to those that had the G/G genotype [OR = 1.89 (1.15-3.11), p = 0.0116]. However, the heterogeneity was very large (*I*^2 ^= 87.8%, p < 0.001; Figure 1). Subgroup analysis showed that in patients with "Non-solid" tumors, there was no significant association between the C/X genotype and cancer [OR = 1.21 (0.95-1.55), p = 0.122; Figure 1], while a significant association was still detected for patients with "Solid" tumors [OR = 2.67 (1.26-5.65), p = 0.0105; Figure 1]. Since even within the "Solid" tumor subgroup, heterogeneity was still very large (*I*^2 ^= 89.8%, p < 0.001), further subgrouping to only those studies dealing with prostate cancer was carried out. When limiting the analysis to only prostate cancer studies, the heterogeneity decreased substantially to low levels (*I*^2 ^= 22.7%, p = 0.250) and the association between the C genotype of the *Mif *promoter polymorphism and cancer was even greater [OR = 3.72 (2.55-5.41), p < 0.0001].

## Discussion

Macrophage migration inhibitory factor is a pro-inflammatory cytokine shown to be upstream of and able to upregulate the production of other inflammatory cytokines [4]. Consequently, MIF is a central modulator of inflammation and a possible link between chronic inflammation and cancer [10]. Several studies have examined the relationship between increased *Mif *mRNA expression and cancer [12-19]. *Mif *promoter polymorphisms that increase *Mif *mRNA expression also result in increased MIF protein production [21,27] and recent studies have established an association between increased MIF serum levels and increased risk for cancer [13,14,32]. The association between high expressing functional *Mif *promoter polymorphisms and prostate cancer appears biologically plausible in view of the documented association between elevated serum MIF protein levels and prostate cancer. MIF's ability to override p53 tumor suppressor activity, sustain MAP (ERK-1/2) kinase phosphorylation and induce the formation of other proinflammatory mediators within the tumor microenvironment [33-36] likely contribute to tumor promotion. The present meta-analysis study is the first, to our knowledge, to critically review published studies on the association between *Mif *promoter polymorphisms and the incidence of cancer. Our meta-analysis focused on the (-173 G/C) *Mif *promoter region polymorphism and cancer since very few studies have examined the association between the number of -794 CATT repeats and cancer. From the 5 studies included in our analysis, our results showed that there is a significant association between having any C allele at the -173 site within the *Mif *promoter and cancer. Because of large heterogeneity in the results, further sub-group analysis showed that there is a strong association between *Mif *promoter genotypes containing a C allele and the development of solid tumors [OR = 2.67 (1.26-5.65), p = 0.0105], while no association was observed in non-solid tumors [OR = 1.21 (0.95-1.55), p = 0.122]. Lastly, analysis of studies dealing only with prostate cancer patients showed the strongest association between cancer incidence and the C allele *Mif *promoter genotype [OR = 3.72 (2.55-5.41)]. Thus, *Mif *promoter genotype may serve as a useful prostate cancer risk marker. 

Some limitations of this meta-analysis study should be considered. First, the number of studies included was low, reflecting the small number of published studies. Second, heterogeneity was large in the global analysis, which justified subgroup analysis but made the number of studies in each subgroup even smaller. Given these two limitations, the link between the C allele *Mif *promoter genotype and prostate cancer awaits validation by future studies. Similarly, whether this link also extends to other solid tumors needs to assessed by future studies. Third, the analysis was based on individual unadjusted OR from each of the studies included, while a better estimate may be obtained by adjusting for potentially contributing factors. For example, Arisawa et al [28] reported an association between the -173C *Mif *allele and gastric cancer only in patients older than 60 years. Therefore, the inclusion of other factors (such as age) may yield a more accurate estimate of the association. Finally, the focus of the current analysis was limited to the *Mif *-173 G/C promoter and did not include -794 CATT repeat polymorphisms due to the very small numbers of studies. Two recent studies described an association between higher MIF producing genotypes (greater number of CATT repeats) and gastric [28] and prostate cancer [27] respectively. These observations should be confirmed by additional studies and the relationship between the two functional *Mif *promoters and carcinogenesis remains to be investigated.

## Conclusions

The current meta-analysis results suggest that the -173C *Mif *promoter polymorphism is associated with an increase in the risk of solid tumor cancer, particularly for prostate cancer. More studies that include a larger variety of cancers are needed to confirm the association of high expressing *Mif *promoter polymorphisms and prostate cancer.

## Methods

### Identification and selection of relevant studies

In order to identify all published articles that examined the association between *Mif *promoter polymorphisms and cancer, a literature search was conducted of the PubMed database through June 2011 using the following MeSH terms and Keywords: 'Macrophage migration inhibitory factor', 'polymorphism' and 'cancer' or 'neoplasm'. Studies were included in the meta-analysis if they met the following criteria: (a) case-control cancer study, (b) cancer diagnosis was confirmed pathologically, and (c) written in English.

### Data extraction

The two investigators independently examined the list of studies for inclusion eligibility, extracted data and reached a consensus on all the items. Meta-analysis was restricted to examining the results of studies dealing with the -173 G/C SNP since few studies examined -794 CATT repeat. The following information was extracted from each eligible study: first author, year of publication, number of cases and controls, *Mif *polymorphisms divided as: GG, any C (CC and CG) for cases and controls, type of cancer: either solid tumor or non-solid tumor.

### Statistical Analysis

The effect of association between *Mif *polymorphism (G/G vs any C; i.e. C/G, C/C) and cancer was calculated as odds ratio (OR) with the corresponding 95% confidence interval [37]. The summary effect was calculated using the Random Effects (RE; Dersimonian and Laird) model [38]. Heterogeneity between studies was assessed using the Q-test and the *I*^2 ^test [39]. Heterogeneity was classified as: low heterogeneity (*I*^2 ^< 25%); moderate (*I*^2 ^= 25-50%) or large (*I*^2 ^> 50%). Heterogeneity was considered statistically significant at p < 0.05. Sub-group analysis further examined the effects in studies investigating non-solid tumors vs. solid tumors. Statistical analysis was conducted using R (version 2.12; http://www.r-project.org) and the meta [40] and metafor [41] packages.

## Competing interests

The authors declare that they have no competing interests.

## Authors' contributions

PLV and KLMS conceived the study, selected the studies to be included, conducted the analysis and prepared the manuscript.
